# A New Valuable Synthesis of Polyfunctionalized Furans Starting from β-Nitroenones and Active Methylene Compounds

**DOI:** 10.3390/molecules24244575

**Published:** 2019-12-13

**Authors:** Elena Chiurchiù, Serena Gabrielli, Roberto Ballini, Alessandro Palmieri

**Affiliations:** Green Chemistry Group, Chemistry Division, School of Science and Technology, University of Camerino, via S. Agostino 1, 62032 Camerino, Italy; elena.chiurchiu@unicam.it (E.C.); serena.gabrielli@unicam.it (S.G.); roberto.ballini@unicam.it (R.B.)

**Keywords:** furans, heterocycles, solid supported species, β-nitroenones, cyclization

## Abstract

Highly functionalized furans are the key scaffolds of many pharmaceuticals and bioactive natural products. Herein, we disclose a new fruitful synthesis of polyfunctionalized furans starting from β-nitroenones and α-functionalized ketones. The protocol involves two steps promoted by solid supported species, and it provides the title targets from satisfactory to very good overall yields and in an excellent diastereomeric ratios.

## 1. Introduction

Furan ring is a useful building block of many biologically active targets, it is the core of many natural compounds and important polymers, and several furan-containing scaffolds serve as privileged structures in medicinal chemistry [1,2,3,4,5,6]. In this context, its significant importance has spurred the scientific community to investigate ever more efficient methodologies for preparing polyfunctionalized furan-based scaffolds, which, in turn, are suitable for further synthetic manipulations. In particular, complex furan derivatives can be achieved by the derivatization of a preexisting furan structure [7,8,9], or by the ex novo ring construction, planning the introduction of specific functionalities in the opportune positions [10,11,12,13]. 

**Figure 1:**
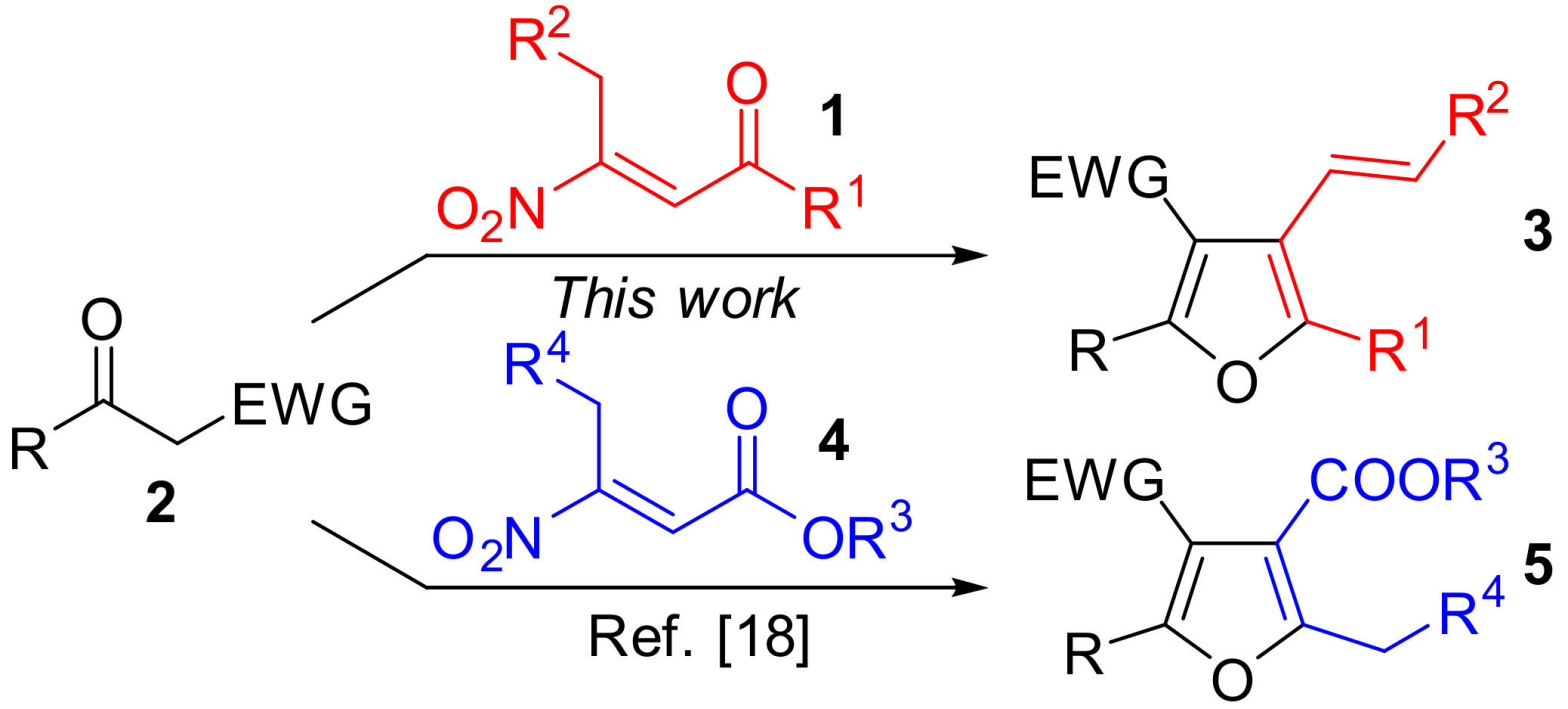
Usage of α-functionalized ketones **2** in combination with β-nitroenones **1** or β-nitroacrylates **4**, for synthesizing tetrasubstituted furans.

Herein, following our studies on the preparation of heteroaromatic systems starting from aliphatic nitro compounds [14,15,16,17], we found β-nitroenones **1** and α-functionalized ketones **2** to be precious and practical building blocks of 3-alkylidene furans **3**. In particular, this study complements our previous research concerning the reaction of α-functionalized ketones **2** with β-nitroacrylates **4** [18] to produce, by a different reaction mechanism, the tetrasubstituted furans **5** (Figure 1). 

**Scheme 1:**
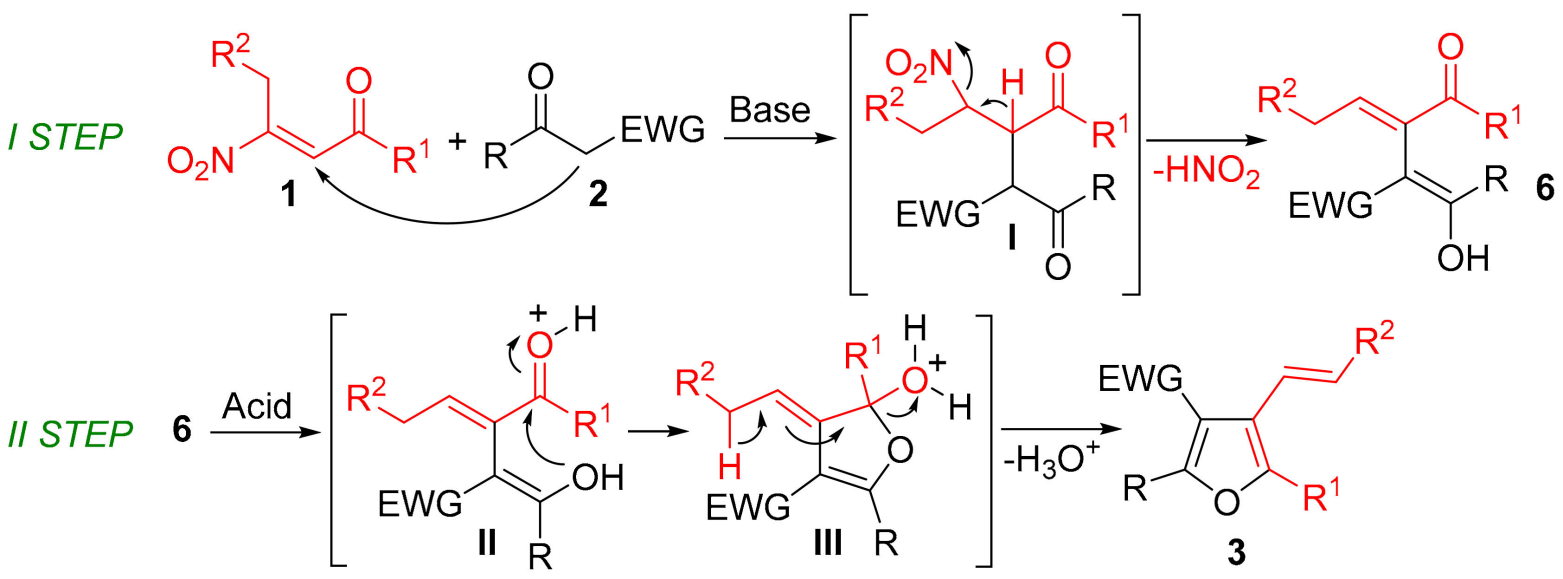
Probable reaction mechanism.

The new protocol involves two steps (Scheme 1): (i) a base promoted addition of **2** to **1** for giving the adduct **I**, which eliminates a molecule of nitrous acid [19], to provide the intermediate **6** and (ii) the acidic catalyzed cyclization of **6** (passing through the adducts **II** and **III**) into the title targets **3**. 

## 2. Results and Discussion

**Scheme 2:**
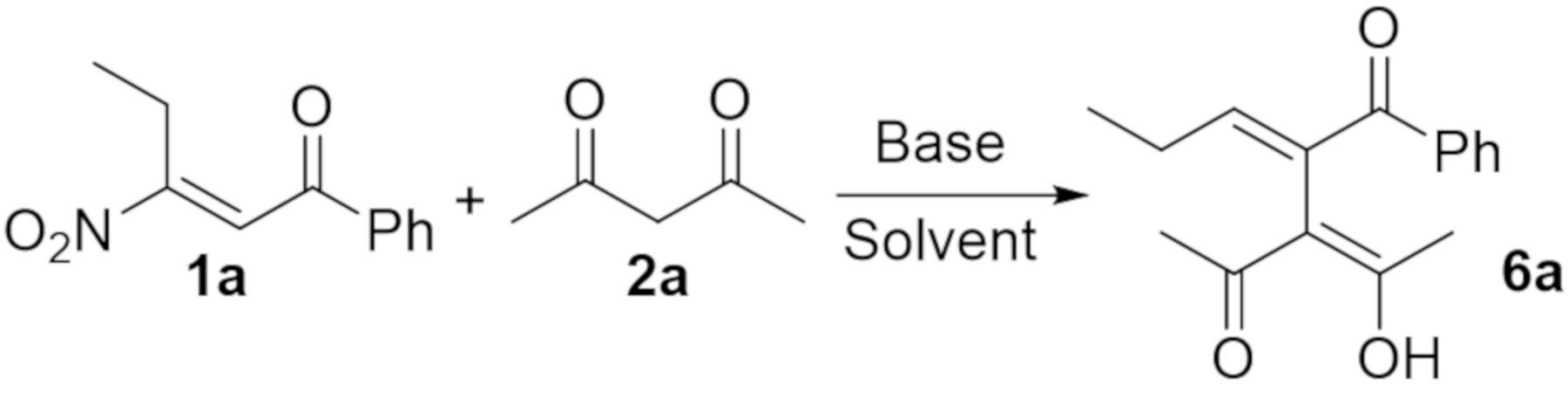
Domino addition–elimination process (I Step).

In our attempt to maximize the process efficacy, we separately studied the two steps. At the beginning, we focused our attention on the domino addition-elimination process (Step I), using, as sample substrates, the β-nitroenone **1a** and diketone **2a** in stoichiometric ratio (Scheme 2).

**Figure 2:**
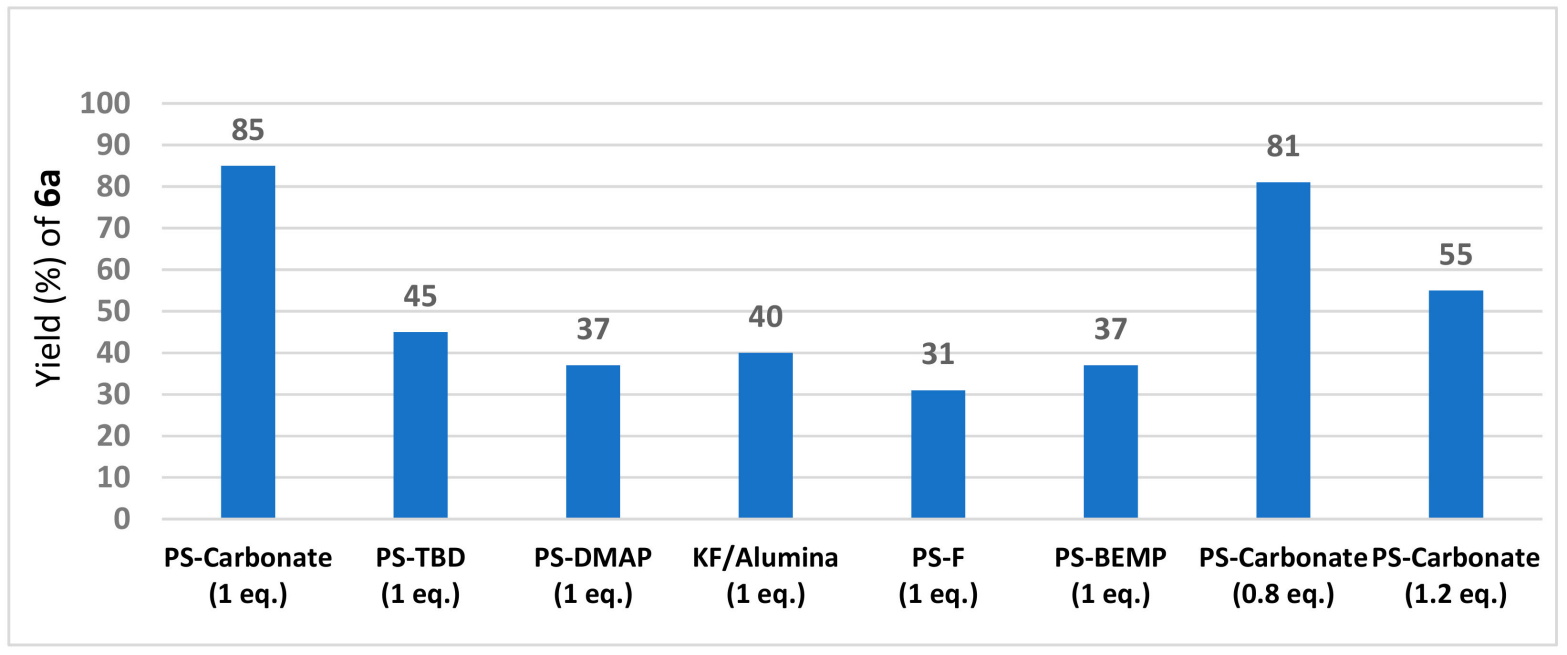
Screening of different supported bases conducting the reaction in MeCN, at room temperature, after two hours (yield of the pure isolated product).

Initially, we tested different supported bases, conducting the reactions in acetonitrile. The best yield of **6a** was obtained after 2 h (complete conversion), using 1 eq. of PS-carbonate at room temperature (Figure 2). 

**Figure 3:**
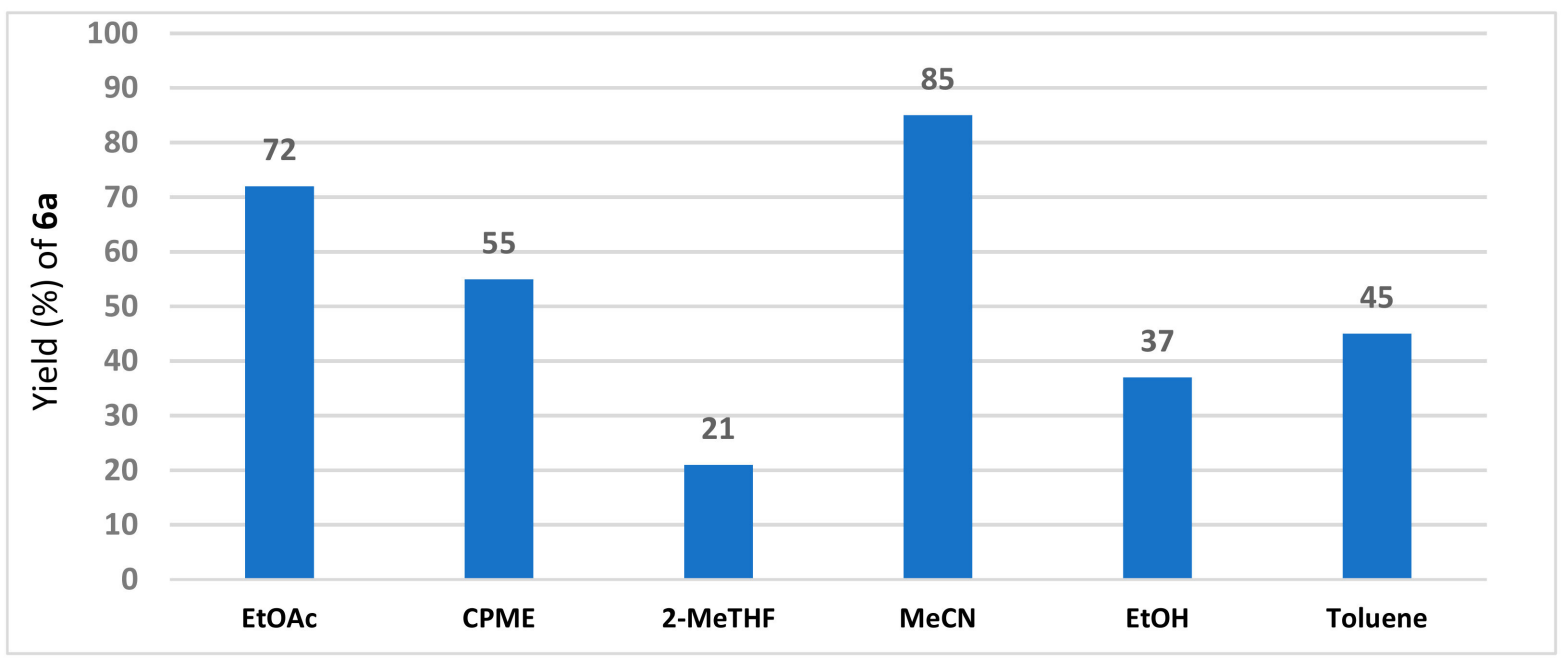
Screening of different solvents conducting the reaction for 2 h, at room temperature and in the presence of 1 eq. of PS-carbonate (yield of the pure isolated product).

Then, once we identified the best base for promoting the one-pot addition-elimination process, we moved our attention to the selection of the reaction media. In this sense, a variety of solvents were screened, and, as depicted in the Figure 3, acetonitrile was the most effective; only ethyl acetate provided **6a** in acceptable yield. 

**Scheme 3:**
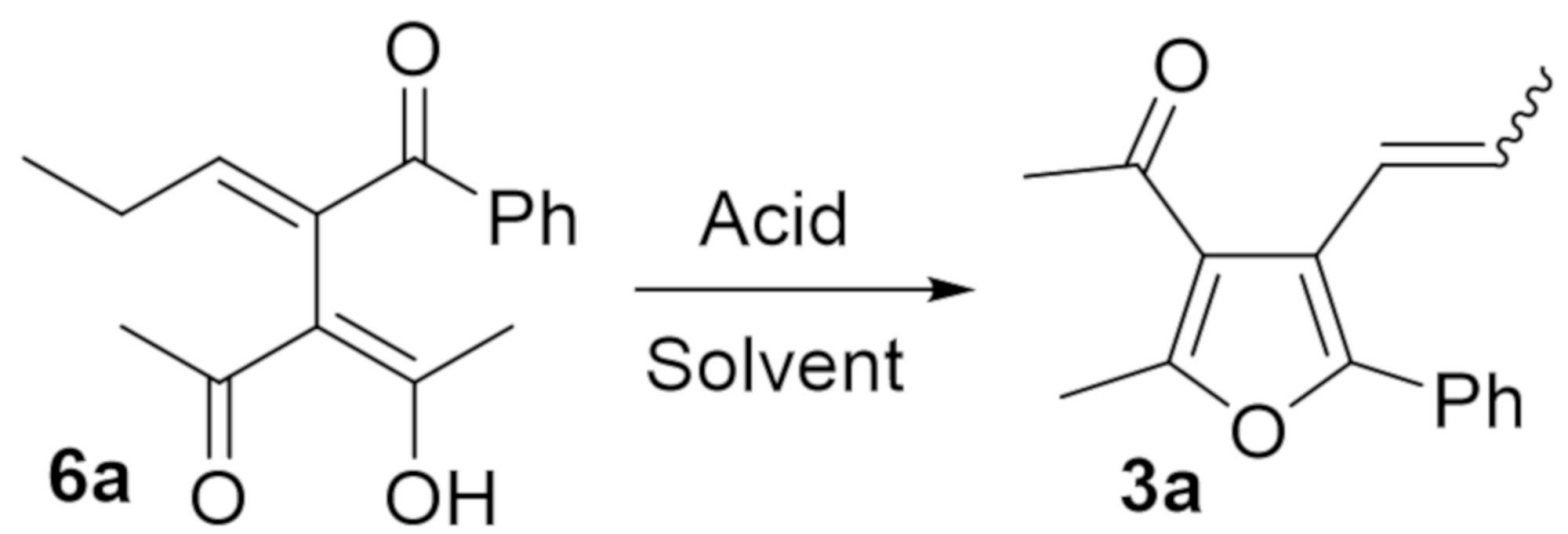
Acidic catalyzed cyclization of **6a** into **3a** (II Step).

After the optimization of the I step (1 eq. of PS-carbonate, MeCN, room temperature, 2 h), we performed an analogous study to optimize the reaction conditions for converting **6a** into the tetrasubstituted furan **3a** (II Step, Scheme 3). In this regard, and based on our experience about the use of Amberlyst 15 for promoting cyclization reactions [11,15,20], we explored this heterogeneous acidic species in different solvents and reaction temperatures (Table 1). 

As reported in Table 1, the use of 0.6 g/mmol of Amberlyst 15 in EtOAc, at 80 °C, produces, after two hours, **6a** in excellent yield and diastereomeric ratio (*Entry i*, 91%, *E*:*Z* = 96:4). After increasing the temperature to 100 °C, the reaction finished in one hour, however the yield and the diastereomeric ratio decreased to 74% and 90:10, respectively. On the other hand, at a lower temperature (60 °C), we observed a longer reaction time (four hours) and a dramatic cutoff of the yield to 45%, albeit the diastereomeric ratio remained unchanged, at 96:4. 

Additional heterogeneous acidic species, such as Montmorillonite K10, acidic alumina, BF_3_/SiO_2_, AlCl_3_/SiO_2_, and Dowex^®^ 50Wx8-200 were also tested. Acidic alumina was completely ineffective (recovered only **6a**); BF_3_ and AlCl_3_ on SiO_2_ provided a complex and inseparable mixture of byproducts, while Montmorillonite K10 and Dowex^®^ provided **3a** in 13% and 24% of yield, respectively.

**Scheme 4:**
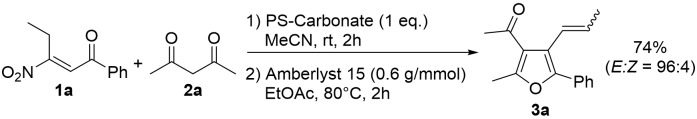
Overall synthesis of **3a**.

Successively, in order to minimize the waste production and the energy consumption, we coupled the two steps, avoiding the purification of the intermediate **6a**. In this aim, after the accomplishment of the I Step, PS-carbonate was removed by filtration, the resin was washed with fresh EtOAc, and then the solvent was evaporated under reduced pressure to give the crude adduct **6a**, which was directly submitted to the II Step, providing **3a** in 74% overall yield (Scheme 4). It is important to note that this result is absolutely comparable with that obtained over the two distinct steps (I Step 85%, II Step 91%, which correspond to a total yield of 77%). 

**Scheme 5:**
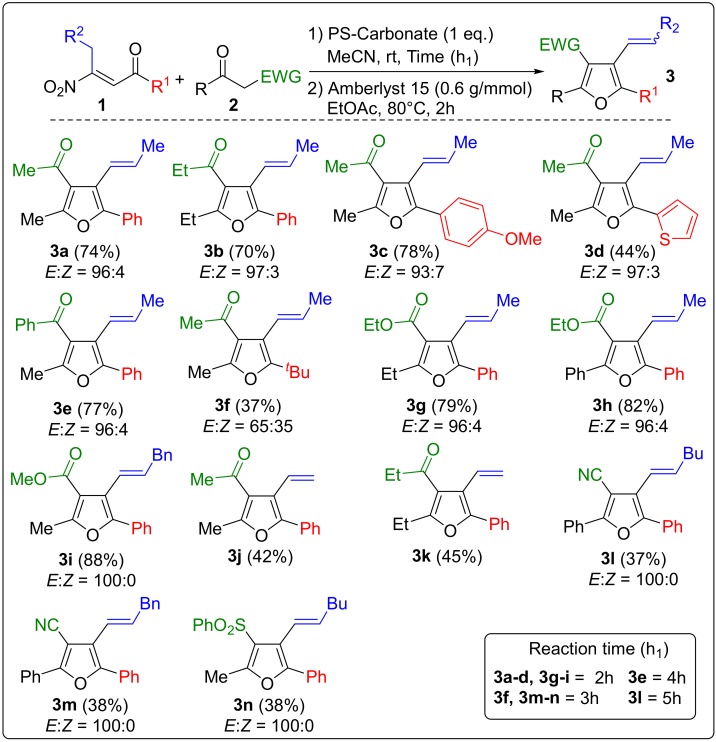
Substrate scope demonstration.

Finally, with the scope to assess the generality of our protocol, we submitted a variety of β-nitroenones **1** and functionalized ketones **2** to the optimized reaction conditions (Scheme 5). Thanks to the mild conditions, it was possible to install a variety of functionalities on the furan ring (ketone, ester, nitrile, and sulfone), obtaining the products **3a**–**n** from satisfactory to very good overall yields (37–88%), and in excellent diastereomeric ratios (*E*:*Z* > 93:7), with the exception of compound **3f** (*E:Z* = 65:35). Furans **3i** and **3l**–**n** were isolated as single *E* diastereoisomer. 

## 3. Conclusions

In conclusion, by exploiting the high reactivity of β-nitroenones, we developed a new general and efficient two-step protocol for synthesizing poly-functionalized furans in good overall yields and excellent diastereoselectivity. In particular, thanks to the mild reaction conditions, a plethora of functional groups can be tolerated, thus giving the possibility to install several functionalities on the furan ring, such as ketone, ester, nitrile, and sulfone. Moreover, since the use of solid supported species in both steps, it was possible to avoid the use of the typical wasteful aqueous work-up, reducing the operation to a simple filtration, with evident advantages from the sustainability viewpoint.

## 4. Materials and Methods

### 4.1. General Section

OXFORD NMR S400, Varian Mercury Plus 400, Oxford, United Kingdom, equipped with workstation Sun Blade 150, software VNMRJ 1.1d, and operating system Solaris 9. ^1^H NMR analyses were recorded at 400 MHz and ^13^C NMR analyses were recorded at 100 MHz. Ir spectra were recorded with a Spectrum Two FT-IR spectrometer, Waltham, MA, United States equipped with ZnSe window, Dynascan Interferometer, detector type LiTaO_3_, and Spectrum 10 software. Microanalyses were performed with a CHNS-O analyzer Model EA 1108 from Fisons Instruments. GS-MS analyses were obtained on an Agilent GC(6850N)/MS(5973N), Stevens Creek Blvd, Santa Clara, CA, United States, EI technique (70 eV), GC/MSD software, and an HP-5MS column, 30 m, Id 0.25 µm, film thichness 0.25 µm. Microwave irradiations were performed by means of a Biotage^®^ Initiator^+^ from Biotage, Uppsala, Sweden. Compound **3j** is known, and its spectroscopic data are in agreement with those reported in the literature [21].

### 4.2. Chemistry Section

General procedure for the preparation of compounds **3a**–**n**: PS-carbonate (0.286 g, 1 mmol) was added to a stirred solution of the appropriate β-nitroenone **1** (1 mmol) and ketone **2** (1 mmol) in acetonitrile (2 mL, 0.5 M), and the resulting solution was stirred at room temperature for the appropriate time (see Scheme 4). Then the resin was filtered off by washing with fresh ethyl acetate (10 mL), and the crude intermediate **6**, obtained after removal of the solvent under reduced pressure, was solubilized in ethyl acetate (12 mL) and treated with Amberlyst 15 (0.6 g) and irradiated Biotage^®^ Initiator^+^, at 80 °C, for 2 h. Finally, the Amberlyst 15 was removed by filtration (washing with fresh ethyl acetate, 10 mL), the solvent evaporated under vacuum, and the crude product **3** obtained from it was purified by flash chromatography column (95:5 hexane/Et_2_O).

*Compound***6a**. Clear oil. IR (cm^−1^, neat): 733, 1597, 1646. ^1^H-NMR (CDCl_3_, 400 MHz) δ: 1.07 (t, 3H, *J* = 7.5 Hz), 2.01 (s, 6H), 2.24 (qui, 2H, *J* = 7.5 Hz), 6.65 (t, 1H, *J* = 7.5 Hz), 7.44–7.50 (m, 2H), 7.54–7.59 (m, 1H), 7.69–7.73 (m, 2H), 15.77 (s, 1H). ^13^C-NMR (100 MHz) δ: 12.9, 23.8, 23.9, 107.9, 128.6, 129.6, 132.2, 136.5, 138.5, 153.2, 191.2, 191.3, 197.0. GC-MS (70 eV): *m/z*: 258 (13), 229 (100), 187 (14), 151 (26), 105 (56), 77 (49), 43 (77). Anal. Calcd. for C_16_H_18_O_3_ (253.32): C, 74.40; H, 7.02. Found: C, 74.44; H, 6.98.

*Compound***3a**. Pale yellow oil. IR (cm^−1^, neat): 693, 764, 950, 1667, 2919. ^1^H-NMR (CDCl_3_, 400 MHz) δ: 1.87 (dd, 3H, *J* = 1.8, 6.6 Hz, CH_3_CH), 2.44 (s, 3H, CH_3_CO), 2.58 (s, 3H, CH_3_), 5.81 (dq, 1H, *J* = 6.6, 15.9 Hz, CHCH_3_), 6.46 (dq, 1H, *J* = 1.8, 15.9 Hz, CH=CH), 7.25–7.29 (m, 1H, *p*-Ph), 7.35–7.40 (m, 2H, *m*-Ph), 7.66–7.69 (m, 2H, *o*-Ph). ^13^C-NMR (100 MHz) δ: 14.7, 19.0, 31.4, 119.3, 121.8, 125.4, 126.4, 127.7, 128.6, 131.1, 132.4, 147.4, 156.8, 196.5. GC-MS (70 eV): *m/z*: 240 (100), 225 (50), 197 (25), 183 (12), 155 (14), 153 (13), 152 (12), 105 (19), 77 (26), 43 (47). Anal. Calcd. for C_16_H_16_O_2_ (240.3): C, 79.97; H, 6.71. Found: C, 80.02; H, 6.75. 

*Compound***3b**. Yellow waxy solid. IR (cm^−1^, neat): 689, 764, 927, 1671, 2939, 2974. ^1^H-NMR (CDCl_3_, 400 MHz) δ: 1.14 (t, 3H, *J* = 7.31 Hz, CH_3_CH_2_CO), 1.29, (t, 3H, *J* = 7.54 Hz CH_3_CH_2_), 1.87 (dd, 3H, *J* = 1.8, 6.6 Hz, CH_3_CH), 2.72 (q, 2H, *J* = 7.3 Hz, CH_2_CO), 2.91 (q, 2H, *J* = 7.5 Hz, CH_2_), 5.77 (dq, 1H, *J* = 6.6, 15.9 Hz, CHCH_3_), 6.46 (dq, 1H, *J* = 1.8, 15.9 Hz, CH=CH), 7.24–7.29 (m, 1H, *p*-Ph), 7.35–7.41 (m, 2H, *m*-Ph), 7.65–7.69 (m, 2H, *o*-Ph). ^13^C-NMR (100 MHz) δ: 8.5, 12.7, 19.0, 21.8, 36.6, 119.0, 122.0, 125.4, 126.4, 127.6, 128.6, 131.2, 132.1, 147.3, 160.6, 200.4. GC-MS (70 eV): *m/z*: 268 ([M^+^], 76), 239 (100), 211 (18), 155 (15), 105 (3), 77 (26), 57 (25), 29 (10). Anal. Calcd. for C_18_H_20_O_2_ (268.36): C, 80.56; H, 7.51. Found: C, 7.55; H, 7.54.

*Compound***3c**. Yellow oil. IR (cm^−1^, neat): 732, 831, 1026, 1176, 1246, 1505, 1667, 2839, 2935. ^1^H-NMR (CDCl_3_, 400 MHz) δ: 1.86 (dd, 3H, *J* = 1.8, 6.6 Hz, CH_3_CH), 2.40 (s, 3H, CH_3_CO), 2.54 (s, 3H, CH_3_), 3.83 (s, 3H, CH_3_O), 5.78 (dq, 1H, *J* = 6.6, 15.9 Hz, CHCH_3_), 6.42 (dq, 1H, *J* = 1.8, 15.9 Hz, CH=CH), 6.87–6.93 (m, 2H, *m*-Ar), 7.57–7.61 (m, 2H, *o*-Ar). ^13^C-NMR (100 MHz) δ: 14.7, 19.0, 31.4, 55.5, 114.1, 117.9, 122.0, 123.8, 126.9, 127.9, 132.0, 147.6, 156.3, 159.2, 196.6. GC-MS (70 eV): *m/z*: 270 ([M^+^], 100), 255 (25), 227 (24), 213 (16), 185 (13), 135 (14), 43 (34). Anal. Calcd. for C_17_H_18_O_3_ (270.33): C, 75.53; H, 6.71. Found: C, 75.48; H, 6.67.

*Compound***3d**. Orange oil. IR (cm^−1^, neat): 835, 950, 1247, 1505, 1667, 2931, 2966. ^1^H-NMR (CDCl_3_, 400 Hz) δ: 1.91 (dd, 3H, *J* = 1.7, 6.6 Hz, CH_3_CH), 2.40 (s, 3H, CH_3_CO), 2.55 (s, 3H, CH_3_), 5.94 (dq, 1H, *J* = 6.6, 15.9 Hz, CHCH_3_), 6.38 (dq, 1H, *J* = 1.8, 15.9 Hz, CH=CH), 7.03 (dd, 1H, *J* = 3.6, 5.1 Hz, CHCH=CH), 7.23 (dd, 1H, *J* = 1.2, 5.1 Hz, CCHCH), 7.32 (dd, 1H, *J* = 1.2, 3.6 Hz, CHS). ^13^C-NMR (100 MHz) δ: 14.8, 19.0, 31.4, 118.8, 121.3, 124.1, 124.4, 124.8, 127.3, 132.7, 133.7, 143.7, 156.9, 196.1. GC-MS (70 eV): *m/z*: 248 ([M+2^+^], 5) 246 ([M^+^], 100), 231 (31), 203 (20), 189 (13), 161 (17), 111 (21), 43 (39). Anal. Calcd. for C_14_H_14_O_2_S (246.32): C, 68.27; H, 5.73; S, 13.02. Found: C, 68.33; H, 5.69; S, 12.98.

*Compound***3e**. Yellow oil. IR (cm^−1^, neat): 693, 764, 906, 1330, 1445, 1596, 1651, 2851, 2914, 3061. ^1^H-NMR (CDCl_3_, 400 MHz) δ: 1.6 (dd, 3H, *J* = 1.7, 6.6 Hz, CH_3_CH), 2.30 (s, 3H, CH_3_), 5.61 (dq, 1H, *J* = 6.6, 15.9 Hz, CHCH_3_), 6.30 (dq, 1H, *J* = 1.7, 15.9 Hz, CH=CH), 7.28–7.33 (m, 1H, *p*-Ph), 7.39–7.47 (m, 4H, *m*-Ph, *m*-PhCO), 7.53–7.58 (m, 1H, *p*-PhCO), 7.65–7.69 (m, 2H, *o*-Ph), 7.84–7.88 (m, 2H, *o*-PhCO). ^13^C-NMR (100 MHz) δ: 13.7, 19.0, 119.9, 120.4, 122.7, 126.6, 127.8, 128.7, 128.8, 129.9, 131.1, 131.4, 133.2, 138.7, 147.8, 154.6, 139.6. GC-MS (70 eV): *m/z*: 302 ([M^+^], 100), 288 (18), 105 (61), 77 (45). Anal. Calcd. for C_21_H_18_O_2_ (302.37): C, 83.42; H, 6.00. Found: C, 83.47; H, 6.04.

*Compound***3f** (Diastereomeric ratio *E:Z* = 65:35). Pale yellow oil. IR (cm^−1^, neat): 633, 835, 950, 1029, 1176, 1247, 1505, 1667, 2930, 2966. ^1^H-NMR (CDCl_3_, 400 MHz) δ: 1.25 (s, 3.15H, (*Z*) *^t^*Bu), 1.28 (s, 5.85H, (*E*) *^t^*Bu), 1.53 (dd, 1.05H, *J* = 1.8, 6.6 Hz, (*Z*) CH_3_CH), 1.83 (dd, 1.95H, *J* = 1.8, 6.6 Hz, (*E*) CH_3_CH), 2.30 (s, 1.05H, (*Z*) CH_3_CO), 2.31 (s, 1.95H, (*E*) CH_3_CO), 2.43 (s, 1.95H, (*E*) CH_3_), 2.47 (s, 1.05H, (*Z*) CH_3_), 5.56 (dq, 0.65H, *J* = 6.6, 15.9 Hz, (*E*) CHCH_3_), 5.81 (dq, 0.35H, *J* = 6.6, 11 Hz, (*Z*) CHCH_3_), 6.29–6.34 (m, 1H, (*E,Z*) CH=CH). ^13^C-NMR (100 MHz) δ:14.4, 14.7, 14.8, 18.7, 29.4, 30.0, 30.1, 31.5, 33.9, 34.1, 113.2, 116.2, 123.0, 123.6, 123.7, 124.0, 130.3, 131.5, 154.5, 155.6, 155.7, 156.1, 196.6, 197.1. GC-MS (70 eV): *m/z*: 220 ([M^+^], 35), 205 (100), 163 (10), 145 (12), 43 (37). Anal. Calcd. for C_14_H_20_O_2_ (220.31): C, 76.33; H, 9.15. Found: C, 76.37; H, 9.18.

*Compound***3g**. Yellow oil. IR (cm^−1^, neat): 693, 764, 1097, 1211, 1707, 2938, 2978. ^1^H-NMR (CDCl_3_, 400 MHz) δ: 1.29 (t, 3H, *J* = 7.5 Hz, CH_3_CH_2_), 1.37 (t, 3H, *J* = 7.1 Hz, CH_3_CH_2_O), 1.83 (dd, 3H, *J* = 1.8, 6.6 Hz, CH_3_CH), 3.00 (q, 2H, *J* = 7.5 Hz, CH_2_), 4.31 (q, 2H, *J* = 7.1 Hz, CH_2_O), 5.94 (dq, 1H, *J* = 6.6, 16.0 Hz, CHCH_3_), 6.50 (dq, 1H, *J* = 1.8, 16.0 Hz, CH=CH), 7.24–7.29 (m, 1H, *p*-Ph), 7.34–7.39 (m, 2H, *m*-Ph), 7.68–7.71 (m, 2H, *o*-Ph). ^13^C-NMR (100 MHz) δ: 12.6, 14.5, 19.1, 21.9, 60.3, 113.9, 119.9, 121.4, 125.4, 126.7, 127.7, 128.6, 130.9, 131.4, 163.1, 164.7. GC-MS (70 eV): *m/z*: 284 ([M^+^], 100), 269 (12), 255 (50), 239 (16), 237 (35), 223 (11), 211 (11), 209 (10), 195 (11), 182 (13), 181 (12), 165 (10), 153 (11), 152 (12), 105 (34), 77 (32). Anal. Calcd. for C_18_H_20_O_3_ (284.35): C, 76.03; H, 7.09. Found: C, 75.98; H, 7.04.

*Compound***3h**. Yellow oil. IR (cm^−1^, neat): 689, 764, 1065, 1223, 1485, 1714, 2910, 2934, 2978. ^1^H-NMR (CDCl_3_, 400 MHz) δ: 1.32 (t, 3H, *J* = 7.1 Hz, CH_3_), 1.88 (dd, 3H, *J* = 7.1 Hz, CH_3_CH), 4.34 (q, 2H, *J* = 7.1 Hz, CH_2_), 6.00 (dq, 1H, *J* = 6.6, 16 Hz, CHCH_3_), 6.51 (dq, 1H, *J* = 1.8, 16 Hz, CH=CH), 7.30–7.46 (m, 6H, *m,p*-Ph), 7.74–7.82 (m, 4H, *o*-Ph). ^13^C-NMR (100 MHz) δ: 14.3, 19.2, 61.2, 115.8, 120.9, 125.7, 126.9, 127.7, 128.1, 128.3, 128.5, 128.8, 129.1, 130.2, 130.9, 148.8, 153.9, 165.5. GC-MS (70 eV): *m/z*: 332 ([M^+^], 100), 285 (33), 269 (25), 215 (13), 105 (57), 77 (29). Anal. Calcd. for C_22_H_20_O_3_ (332.40): C, 79.50; H, 6.07. Found: C, 79.47; H, 6.03.

*Compound***3i**. Yellow waxy solid. IR (cm^−1^, neat) 693, 760, 966, 1093, 1211, 1441, 1707, 2851, 2907, 2950, 3025. ^1^H-NMR (CDCl_3_, 400 MHz) δ: 2.61 (s, 3H, CH_3_), 3.53 (dd, 2H, *J* = 1.4, 7.1 Hz, CH_2_), 3.81 (s, 3H, CH_3_O), 6.07 (dt, 1H, *J* = 7.1, 16 Hz, CHBn), 6.59 (dt, 1H, *J* = 1.6, 16 Hz, CH=CH), 7.20–7.35 (m, 8H, *m,p*-Ph, *m*,*o*,*p*-PhCH_2_), 7.64–7.68 (m, 2H, *o*-Ph). ^13^C-NMR (100 MHz) δ: 14.6, 40.0, 51.4, 114.6, 119.5, 121.6, 126.32, 126.8, 127.8, 128.6, 128.7, 128.7, 131.0, 134.2, 140.1, 148.0, 158.8, 156.2. GC-MS (70 eV): *m/z*: 332 ([M^+^], 100), 300 (100), 284 (40), 257 (22), 241 (19), 230 (29), 223 (44), 209 (19), 182 (9), 165 (10), 152 (15), 115 (15), 105 (29), 91 (29), 77 (33), 43 (14). Anal. Calcd. for C_22_H_20_O_3_ (332.40): C, 79.50; H, 6.07. Found: C, 79.54; H, 6.10.

*Compound***3j**. Clear oil. IR (cm^−1^, neat) 665, 693, 768, 950, 1069, 1132, 1390, 1667, 2922, 2954. ^1^H-NMR (CDCl_3_, 400 MHz) δ: 2.44 (s, 3H, CH_3_CO), 2.57 (s, 3H, CH_3_), 5.41 (dd, 1H, *J* = 1.7, 24.4 Hz, CH), 5.44 (dd, 1H, *J* = 1.7, 17.7 Hz, CH), 6.84 (dd, 1H, *J* = 11.2, 17.7 Hz, CH=CH_2_), 7.27–7.32 (m, 1H, *p*-Ph), 7.35–7.41 (m, 2H, *m*-Ph), 7.66–7.70 (m, 2H, *o*-Ph). ^13^C-NMR (100 MHz) δ: 14.7, 31.5, 119.5, 120.7, 124.2, 126.7, 128.0, 128.7, 128.8, 130.7, 148.0, 156.8, 196.3. GC-MS (70 eV): *m/z*: 226 ([M^+^], 100), 225 (77), 211 (22), 183 (40), 165 (15), 155 (23), 141 (22), 115 (19), 105 (18), 77 (25), 43 (41). Anal. Calcd. for C_15_H_14_O_2_ (226.27): C, 79.62; H, 6.24. Found: C, 79.67; H, 6.20.

*Compound***3k**. Yellow waxy solid. IR (cm^−1^, neat): 693, 768, 926, 1671, 2938, 2978. ^1^H-NMR (CDCl_3_, 400 MHz) δ: 1.14 (t, 3H, *J* = 7.3 Hz, CH_3_CH_2_CO), 1.30 (t, 3H, *J* = 7.5 Hz, CH_3_CH_2_), 2.75 (q, 2H, *J* = 7.3 Hz, CH_2_CO), 2.91 (q, 2H, *J* = 7.5, CH_2_), 5.35 (dd, 1H, *J* = 1.7, 17.7 Hz, CH), 5.41 (dd, 1H, *J* = 1.7, 11.2 Hz, CH), 6.83 (dd, 1H, *J* = 11.2, 17.7 Hz, CH=CH_2_), 7.27–7.32 (m, 1H, *p*-Ph), 7.36–7.41 (m, 2H, *m*-Ph), 7.65–7.70 (m, 2H, *o*-Ph). ^13^C-NMR (100 MHz) δ: 8.5, 12.8, 21.7, 36.7, 119.2, 120.3, 123.1, 126.7, 127.9, 128.7, 128.7, 130.9, 147.9, 160.5, 200.3. GC-MS (70 eV): *m/z*: 254 ([M^+^], 100), 253 (40), 235 (20), 225 (68), 197 (23), 141 (27), 115 (13), 105 (50), 77 (29), 57 (23), 29 (10). Anal. Calcd. for C_17_H_18_O_2_ (254.33): C, 80.28; H, 7.13. Found: C, 80.24; H, 7.10.

*Compound***3l**. Pale yellow waxy. IR (cm^−1^, neat): 685, 764, 962, 1489, 2225, 2859, 2926, 2954. ^1^H-NMR (CDCl_3_, 400 MHz) δ: 0.95 (t, 3H, *J* = 7.2 Hz, CH_3_), 1.37–1.56 (m, 4H, CH_3_CH_2_CH_2_), 2.25–2.32 (m, 2H, CH_2_CH), 6.43 (dt, 1H, *J* = 1.5, 16.1 Hz, CH=CH), 6.63 (dt, 1H, *J* = 6.9, 16.1 Hz, CHBu), 7.37–7.53 (m, 6H, *m,p*-Ph, *m,p*-Ph), 7.66–7.70 (m, 2H, *o*-Ph), 8.05–8.09 (m, 2H, *o*-Ph). ^13^C-NMR (100 MHz) δ: 14.2, 22.5, 31.4, 33.6, 93.0, 115.9, 117.9, 121.0, 125.8, 127.0, 128.3, 128.8, 129.0, 129.3, 129.8, 130.3, 136.8, 149.0, 159.0. GC-MS (70 eV): *m/z*: 327 ([M^+^], 100), 284 (34), 250 (29), 206 (10), 105 (48), 77 (33). Anal. Calcd. for C_23_H_21_NO (327.43): C, 84.37; H, 6.46; N, 4.28. Found: C, 84.41; H, 6.42; N, 4.31.

*Compound***3m**. Yellow waxy solid. IR (cm^−1^, neat): 671, 691, 766, 1491, 2231, 2924, 3023, 3058. ^1^H-NMR (CDCl_3_, 400 MHz) δ: 3.63 (dd, 2H, *J* = 1.2, 6.7 Hz, CH_2_), 6.46 (dt, 1H, *J* = 1.6, 16.1 Hz, CH=CH), 6.82 (dt, 1H, *J* = 6.7, 16.1 Hz, CHBn), 7.17–7.54 (m, 11H, *m,o,p*-PhCH_2_, *m,p*-Ph, *m,p*-Ph), 7.62 (m, 2H, *o*-Ph), 8.04–8.09 (m, 2H, *o*-Ph). ^13^C-NMR (100 MHz) δ: 39.9, 92.8, 115.8, 119.5, 120.6, 125.8, 126.6, 127.0, 128.2, 128.8, 128.9 (2C), 129.0, 129.3, 129.6, 130.4, 134.5, 139.5, 149.5, 159.2. GC-MS (70 eV): *m/z*: 361 ([M^+^], 100), 360 (89), 270 (14), 269 (13), 105 (29), 77 (26). Anal. Calcd. for C_26_H_19_NO (361.44): C, 86.40; H, 5.30; N, 3.88. Found: C, 86.45; H, 5.33; N, 3.84.

*Compound***3n**. Yellow oil. IR (cm^−1^, neat): 558, 605, 685, 1160, 1318, 1445, 2859, 2926, 2958. ^1^H-NMR (CDCl_3_, 400 MHz) δ: 0.9 (t, 3H, *J* = 7.2 Hz, CH_3_), 1.23–1.37 (m, 4H, CH_3_CH_2_CH_2_), 2.06–2.12 (m, 2H, CH_2_CH), 2.73 (s, 3H, CH_3_), 5.78 (dt, 1H, *J* = 7.7, 16.1 Hz, CHBu), 6.29 (dt, 1H, *J* = 1.6, 16.1, CH=CH), 7.24–7.59 (m, 6H, *m,p*-Ph, *m,p*-PhS), 7.62–7.66 (m, 2H, *o*-Ph), 7.86–7.94 (m, 2H, *o*-PhS). ^13^C-NMR (100 MHz) δ: 14.0, 14.2, 22.5, 31.1, 33.2, 118.1, 122.8, 126.7, 127.3, 128.0, 128.2, 128.6, 129.1, 130.3, 133.2, 138.7, 142.9, 148.2, 156.9. GC-MS (70 eV): *m/z*: ([M+2^+^], 7), 380 ([M^+^], 100), 232 (31), 311 (69), 196 (36), 182 (53), 153 (25), 125 (15), 105 (73), 77 (75), 43 (29). Anal. Calcd. for C_23_H_24_O_3_S (380.50): C, 72.60; H, 6.36; S, 8.43. Found: C, 72.65; H, 6.39; S, 8.39.

## Figures and Tables

**Table 1:** Optimization studies of the II Step: conversion of **6a** into **3a**.

Entry	Amberlyst 15 (g/mmol)	Solvent	Temperature (°C) ^1^	Time (h)	Yield (%) of 3a ^2^	*E*:*Z*
*a*	1	MeCN	80	2	53	96:4
*b*	1	EtOAc	80	2	76	96:4
*c*	1	Toluene	80	2	68	82:18
*d*	1	2-MeTHF	80	2	58	90:10
*e*	1	EtOAc	100	1	74	90:10
*f*	1	EtOAc	60	4	45	96:4
*g*	1.2	EtOAc	80	2	82	96:4
*h*	0.8	EtOAc	80	2	89	96:4
*i*	0.6	EtOAc	80	2	91	96:4
*j*	0.4	EtOAc	80	2	85	96:4

^1^ Reaction performed under microwave irradiations in a sealed vessel. ^2^ Yield of the pure isolated product.

## Abbreviations

PS-carbonate	carbonate on polymer support (Sigma-Aldrich code: 21850: loading: 3.5 mmol/g)
PS-DMAP	4-(dimethylamino)pyridine, polymer-bound (Sigma-Aldrich code: 39410, loading: 3 mmol/g)
PS-BEMP	2-tert-butylimino-2-diethylamino-1,3-dimethylperhydro-1,3,2-diazaphosphorine, polymer-bound (Sigma-Aldrich code: 20026, loading: 2.2 mmol/g)
PS-TBD	1,5,7-Triazabicyclo[4.4.0]dec-5-ene bound to polystyrene (Sigma-Aldrich code: 01961, loading: 3.0 mmol/g)
PS-F	fluoride on polymer support (Sigma-Aldrich code: 47060, loading: 3.0 mmol/g)
KF/alumina	potassium fluoride on aluminum oxide (Sigma-Aldrich code: 60244, loading: 5.5 mmol/g)
